# CT image denoising methods for image quality improvement and radiation dose reduction

**DOI:** 10.1002/acm2.14270

**Published:** 2024-01-19

**Authors:** Rabeya Tus Sadia, Jin Chen, Jie Zhang

**Affiliations:** ^1^ Department of Computer Science University of Kentucky Lexington Kentucky USA; ^2^ Department of Medicine‐Nephrology University of Alabama at Birmingham Birmingham Alabama USA; ^3^ Department of Radiology University of Kentucky Lexington Kentucky USA

**Keywords:** CT image, deep learning, denoising, radiation dose reduction

## Abstract

With the ever‐increasing use of computed tomography (CT), concerns about its radiation dose have become a significant public issue. To address the need for radiation dose reduction, CT denoising methods have been widely investigated and applied in low‐dose CT images. Numerous noise reduction algorithms have emerged, such as iterative reconstruction and most recently, deep learning (DL)‐based approaches. Given the rapid advancements in Artificial Intelligence techniques, we recognize the need for a comprehensive review that emphasizes the most recently developed methods. Hence, we have performed a thorough analysis of existing literature to provide such a review. Beyond directly comparing the performance, we focus on pivotal aspects, including model training, validation, testing, generalizability, vulnerability, and evaluation methods. This review is expected to raise awareness of the various facets involved in CT image denoising and the specific challenges in developing DL‐based models.

## 1 INTRODUCTION

Computed tomography (CT) is a workhorse within the radiology community. It provides fine details with speed and clarity. However, for CT, x‐rays must be used to reconstruct image slices of the target region. The ionizing radiation could put patients at risk for the negative effects of x‐ray. The widespread adoption of CT imaging has heightened public awareness of radiation exposure risks. To mitigate these concerns, there has been a substantial effort to develop and implement CT denoising techniques. These methods are essential for reducing radiation doses while concurrently enhancing the quality of images obtained from low‐dose CT scans, thus improving their diagnostic value.

We performed a literature search on publications up to the end of 2022 from PubMed. The use of the keyword “CT” and “image denoising” returned 484 results. We screened the publications to exclude 250 unrelated papers, 9 review papers, and 3 publications that were not in English, leaving a total of 222 publications. The 222 publications were further categorized into three categories: traditional CT denoising methods (65 publications), deep learning (DL)‐based CT denoising methods (99 publications), and application and evaluation studies (58 publications).

The nine review papers importantly covered three topics: traditional image denoising methods,^1^, ^2^, ^3^ DL‐based image denoising methods^4^, ^5^, ^6^, ^7^, ^8^ as well as its application.^9^ With rapid advances in Artificial Intelligence (AI) techniques, we feel there is a need to provide a more comprehensive review including most recently developed methods. Beyond direct comparison of the performance, most important, we will discuss model training, validation and testing, generalizability and vulnerability, and evaluation methods. To the best of our knowledge, there is no such a review that examines those challenges in developing DL‐based models. Hence, we provide a review regarding this topic based on a thorough analysis of existing literature.

## 2 IMAGE NOISE AND NOISE REDUCTION

There are various reasons that generate the noise in CT images, including but not limited to radiation dose, electronic noise, slice thickness, and patient size. Radiation dose plays a crucial role in determining the noise level in CT images. Increasing the radiation dose can lead to a reduction in noise, which improves image clarity. However, this comes with the drawback of higher radiation exposure. It is important to optimize the settings of the examination to achieve a balance between reducing radiation dose and preserving sufficient image quality for accurate diagnostic interpretation.

**FIGURE 1:**
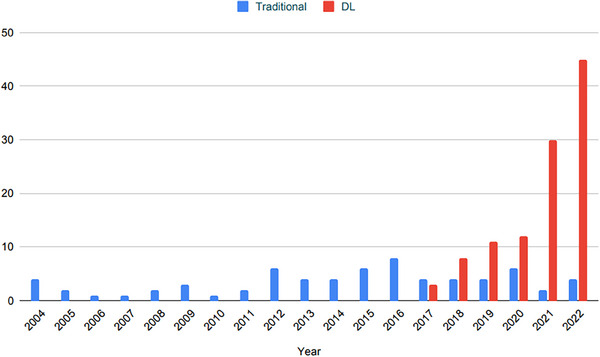
Annual number of publications for traditional and DL‐based CT denoising methods (accessed on December 2022).

Noise reduction is the process of removing noise from a signal. There are many noise reduction algorithms in image processing, such as nonlocal means,^2^ wavelet transform,^10^ well adopted iterative reconstruction,^3^ and most recently, DL‐based approaches. In this review, we categorize all image denoising methods beyond DL approaches as traditional methods. Figure 1 shows the annual number of publications for traditional and DL‐based CT denoising methods.

While the magnitude of noise in an image is a focus, noise texture is also important because it can have a strong effect on how well structures can be visualized. This raises a question on how to evaluate the performance of image denoising algorithms.

## 3 TRADITIONAL NOISE REDUCTION METHODS

As our main emphasis is not on traditional noise reduction methods, we present a concise overview to maintain the overall integrity of the topic.

Filtered back projection (FBP) has traditionally been the standard method for CT image reconstruction, however, iterative reconstruction (IR) has now become widely adopted in clinical practice. IR turns the FBP process into an algorithm that operates iteratively. Compared to FBP, IR can handle more complex imaging scenarios, such as dealing with noisy or incomplete data, or accommodating non‐uniformly sampled data. Another advantage of IR is that it produces images with higher spatial resolution and better image quality in some cases, particularly when dealing with low‐dose or sparse data. This is because iterative algorithms can use information from neighboring pixels to improve the estimate of each pixel's value, resulting in less noise and better detail in the reconstructed image.

There are several CT image denoising algorithms, for example, wavelet‐based denoising,^11^ non‐local means denoising,^12^ total variation denoising,^13^ dictionary learning‐based denoising,^14^ and Block‐matching and 3D filtering.^15^

Wavelet‐based denoising is a popular method for reducing noise in CT images. This method involves decomposing the image into different frequency bands using wavelet transforms and then filtering out the noise in each frequency band before reconstructing the image.^11^, ^16^, ^17^, ^18^, ^19^ Non‐local means^12^ denoising is a patch‐based denoising method that compares patches of similar texture or structure across the image to estimate the noise level in each pixel. This method can effectively reduce noise while preserving image details. Total variation^13^ denoising is a variational method that aims to minimize the total variation of the image while preserving its features. This method can reduce noise while preserving edges and sharp features in the image. Dictionary learning‐based^14^ denoising is a machine learning‐based approach that learns a dictionary of image patches from a set of training images and uses this dictionary to denoise new images. This method can effectively reduce noise while preserving image details and structures. Block‐matching and 3D filtering (BM3D)^15^ is an algorithm that involves two major steps: (1) a hard‐thresholding estimation of the original image; (2) a step using the original image, the estimation from step (1), and Wiener filtering. Both steps include grouping, collaborative filtering, and aggregation. The hard‐thresholding and Wiener filtering are both done in the collaborative filtering sub steps.

A detailed review regarding traditional CT image denoising methods, besides IR, was given by Diwakar and Kumar,^1^ while the strengths and weaknesses of IR algorithms were reviewed by Mohammadinejad et al.^3^ Based on our survey of the PubMed, there are 65 publications up to 2022, with the earliest one from 2004. Table 1 provides a distribution of traditional denoising methods in CT images.

**TABLE 1:** Traditional CT noise reduction methods.

	Denoising methods	Number of studies
Spatial domain	Linear and non‐linear filters	13^20^, ^21^
	Variation methods	11^13^, ^22^, ^23^, ^24^, ^25^, ^26^, ^27^, ^28^, ^29^, ^30^, ^31^
	Dictionary learning method	7^32^, ^33^, ^34^, ^35^, ^36^, ^37^, ^38^
	Bilateral and non‐local means filters	13^2^, ^12^, ^39^, ^40^, ^41^, ^42^, ^43^, ^44^, ^45^, ^46^, ^47^, ^48^, ^49^
Transform domain	Wavelet based denoising	5^11^, ^16^, ^17^, ^18^, ^50^
	Threshold estimation	2^10^, ^51^
	Shrinkage rules	2^17^, ^19^
	Intra and inter scale dependencies based denoising	2^52^, ^53^
	Image denoising based on extended versions of transform	6^14^, ^21^, ^51^, ^54^, ^55^, ^56^
	Block‐matching and 3D filtering (BM3D)	4^15^, ^25^, ^57^, ^58^

## 4 DL‐BASED NOISE REDUCTION METHODS

DL techniques have shown great potential in image reconstruction and restoration tasks, outperforming commercial IR algorithm methods in CT denoising. There are several DL approaches for CT image denoising, including but not limited to: convolutional neural networks (CNNs), Generative Adversarial Networks (GANs), Variational Autoencoders (VAEs), Deep Residual Networks (ResNets), Transformer‐based methods, Attention‐based Networks, as well as hybrid approaches that combine multiple approaches, such as CNNs and GANs, to improve the quality and accuracy of CT image denoising.

**FIGURE 2:**
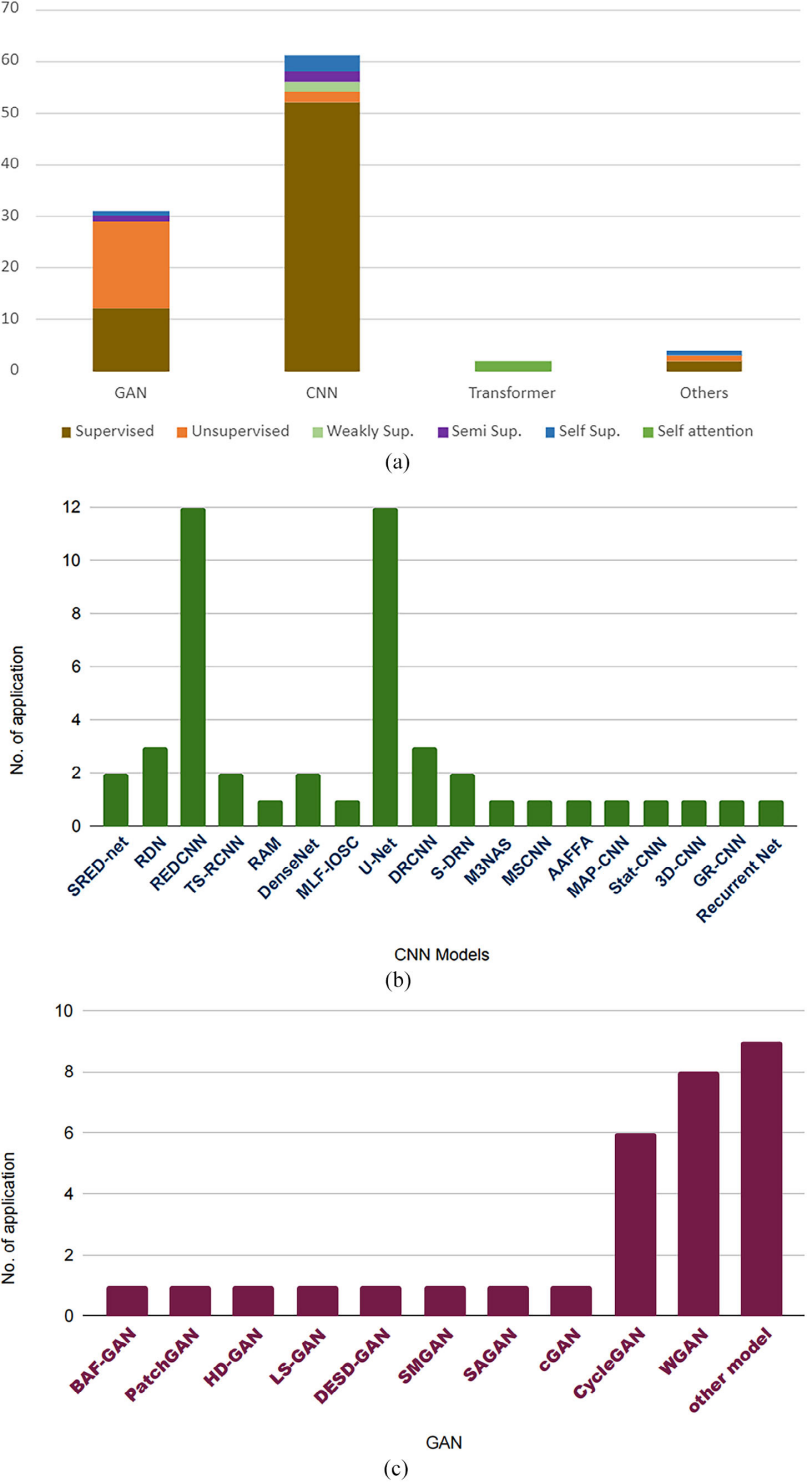
The distribution of deep learn (DL) approaches for CT image denoising: (a) various DL approaches for CT image denoising; (b) models based on convolutional neural networks (CNNs); (c) models based on generative adversarial networks (GANs).

The predominant DL models for CT denoising are GANs and CNNs. As shown in Figure 2a, out of 99 publications examined, 61 studies use the models based on CNN,^59^, ^60^, ^61^, ^62^, ^63^, ^64^, ^65^, ^66^, ^67^, ^68^, ^69^, ^70^, ^71^, ^72^, ^73^, ^74^, ^75^, ^76^, ^77^, ^78^, ^79^, ^80^, ^81^, ^82^, ^83^, ^84^, ^85^, ^86^, ^87^, ^88^, ^89^, ^90^, ^91^, ^92^, ^93^, ^94^, ^95^, ^96^, ^97^, ^98^, ^99^, ^100^, ^101^, ^102^, ^103^, ^104^, ^105^, ^106^, ^107^, ^108^, ^109^, ^110^, ^111^, ^112^, ^113^, ^114^, ^115^, ^116^, ^117^, ^118^, ^119^ while 30 studies are based on GAN.^120^, ^121^, ^122^, ^123^, ^124^, ^125^, ^126^, ^127^, ^128^, ^129^, ^130^, ^131^, ^132^, ^133^, ^134^, ^135^, ^136^, ^137^, ^138^, ^139^, ^140^, ^141^, ^142^, ^143^, ^144^, ^145^, ^146^, ^147^, ^148^, ^149^ Additionally, two studies adopt Transformer‐based approaches.^150^, ^151^ with the remaining approaches including filter‐based methods as well as hybrid methods.^152^, ^153^, ^154^, ^155^, ^156^, ^157^ To be noted that some models are originally developed,^61^, ^62^, ^65^, ^66^, ^68^, ^70^, ^72^, ^73^, ^77^, ^122^, ^153^ while some are developed by modifying original models through modifying loss functions, or layers, or extending original models to different domains.^60^, ^63^, ^64^, ^69^, ^71^, ^72^, ^74^, ^75^, ^76^, ^78^, ^82^, ^84^, ^102^, ^121^, ^123^, ^124^, ^125^, ^147^,^150^, ^151^, ^152^, ^155^, ^158^

### 4.1 Convolutional neural networks (CNNs)

CNN‐based CT denoising methods use a network architecture consisting of multiple layers of convolutional filters to learn features from the noisy input images and produce denoised output images. A traditional CNN involves both convolutional layers and fully connected (FC) layers. The FC layers are the “neurons” in neural networks and do not contain convolutions and instead rely on linear transformations of the input data. CNNs generally utilize supervised training. The convolutional portions of CNNs are also trained to produce the most optimal kernels.

**FIGURE 3:**
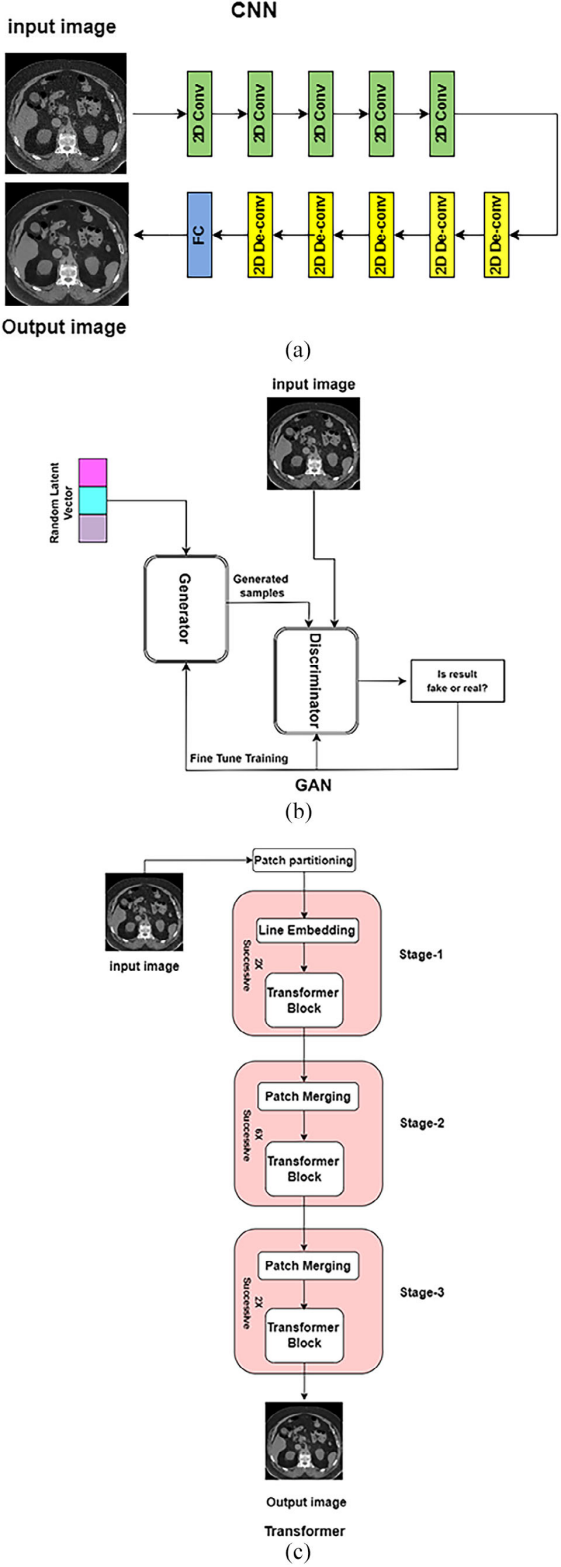
Basic deep learning architectures. (a) CNN. FC: fully connected, (b) GAN, (c) transformer.

Figure 3a shows the basic CNN architecture. In this network, each convolutional layer contributes to noise reduction in CT images by progressively extracting and refining features pertinent to the underlying anatomical structures. It leverages local information through receptive fields, applies non‐linear transformations to discern noise from actual patterns, and learns adaptive filters that diminish noise while preserving critical details. The hierarchical depth of CNN architectures enables the capture of intricate information, facilitating noise suppression, and overall enhancement of CT image quality.

One of the main advantages of CNNs in CT image denoising is their ability to perform automatic feature extraction. CNNs can automatically extract relevant features from the image they are given, which eliminates the need for manual feature engineering.^59^ Another advantage of CNNs is their ability to handle high‐dimensional data. CT images are high‐dimensional and can have a large number of pixels. CNNs are designed to handle this type of information and can process large amounts of data quickly.^159^ Finally, CNNs are known to achieve good performance in a wide range of image processing tasks, including CT image denoising.^79^

Despite their advantages, there are also some potential drawbacks to using CNNs in CT image denoising. One of the main challenges is the need for a significant amount of labeled data to train the network. In most cases, training a CNN requires a large amount of labeled data.^140^ When dealing with CT image denoising, it can be difficult to generate paired noisy and noise‐free images for comparison. Another potential disadvantage of CNNs is their black box nature. CNNs are sometimes considered to be black box models because it can be challenging to understand how they generate their predictions.^90^ This can be a disadvantage in applications where interpretability is important. Finally, CNNs have several hyperparameters that need to be tuned, such as the number of layers, filter sizes, and learning rate. Tuning these hyperparameters can be a time‐consuming process, and the best values may vary depending on the dataset and the specific problem being addressed.

Figure 2b shows the studies using various CNN‐based models. RED‐CNN and U‐NET are the most widely used models. Both RED‐CNN and U‐NET architectures are well‐suited for image‐to‐image translation tasks like those from CT denoising. They feature encoder‐decoder structures that enable effective feature extraction and information preservation through skip connections. Medical images like CT can vary significantly in terms of noise levels, contrast, and structures. RED‐CNN and U‐NET, with their learned representations and feature extraction capabilities, can adapt well to these variabilities.

### 4.2 Generative adversarial networks (GANs)

GANs are a type of DL model that consists of two neural networks: a generator network and a discriminator network. These networks are trained together in an adversarial manner to produce realistic and denoised output images from noisy input images. The generator network creates new data, while the discriminator network evaluates whether the generated data are real or fake. During training, the two networks are trained simultaneously, with the generator trying to produce data, that is, indistinguishable from the real data, and the discriminator trying to correctly identify whether the data are real or fake. Since the generator only needs to minimize a loss function that the discriminator would try to maximize, it does not need to be anything specific. The same is true for the discriminator. Figure 3b depicts the basic GAN architecture.

The use of GANs for CT image denoising has several advantages. First, GANs can produce high‐quality images that are not only visually realistic but also feature fine‐grained details.^132^ Second, GANs are effective at eliminating noise from CT images while preserving the essential characteristics and structures of the images.^143^ Third, GANs are able to understand intricate patterns and correlations in CT scans, which conventional image denoising approaches might struggle to do.

However, GANs can be computationally intensive and require a substantial amount of input data to produce accurate predictions.^146^ If the training data are not representative of the real world or if the model is not trained correctly, GANs have a higher propensity to produce artifacts and errors in the images that they generate.^123^ Additionally, GANs could generate images that are visually realistic but clinically inaccurate, leading to incorrect diagnoses or treatments. As such, GANs are best suited for image processing and computer vision applications, while tasks such as image classification, object detection, and image segmentation are particularly well‐suited to convolutional neural networks (CNNs).

Figure 2c shows the studies using various GAN‐based models. CycleGAN and WGAN are well adopted. CycleGAN is a type of GAN that involves two generators and two discriminators.^122^, ^128^, ^136^, ^142^, ^143^ It is designed to learn the mapping between two domains without the need for paired data. In the context of CT image denoising, CycleGAN can be used to learn the mapping between noisy and clean CT images, enabling it to generate denoised images from noisy input images. One advantage of CycleGAN is that it can be trained on unpaired data, which can make it easier to obtain training data.

WGAN, on the other hand, is a variant of GANs that utilizes a Wasserstein distance metric to measure the distance between the generated and real image distributions.^129^, ^132^, ^133^, ^134^, ^137^, ^140^, ^141^, ^148^ This helps to stabilize the training process and can result in more realistic output images. WGAN has been used for CT image denoising, and has shown promising results in reducing noise and preserving image details.

### 4.3 Transformer‐based methods

Transformers are a type of DL model that were initially developed for natural language processing tasks but have recently gained attention in computer vision due to their ability to model long‐range dependencies between pixels in an image. It is grounded in a self‐attention mechanism that allows it to record dependencies between distinct parts of the input sequence in a non‐linear fashion, which is different from the linear processing of sequential data by conventional neural networks. The Transformer model can be modified for use in the context of CT image denoising by processing high‐dimensional CT pictures in a way that captures spatial dependencies between different regions of the image. To denoise an image using a transformer, the model first processes the input image to extract and analyze key features and patterns. The model then uses this information to identify and separate the noise from the useful signal in the image before applying a denoising algorithm to remove the noise and produce a denoised output image. Overall, transformer‐based CT denoising methods have shown promising results in reducing noise while preserving image details, especially in low‐dose CT scans, by leveraging the power of self‐attention mechanisms to better analyze and process the input data.

Figure 3c shows the basic transformer architecture. By utilizing the self‐attention mechanism, a transformer DL model denoises CT images by capturing contextual information and long‐range dependencies and adaptively weights input characteristics to distinguish between meaningful structures and noise. The model can simultaneously focus on different areas of the image utilizing the multi‐head attention mechanism, which helps the model understand complex relationships and patterns in the data to effectively reduce noise.

One of the advantages of the Transformer model in CT image denoising is its ability to capture spatial dependencies. The model's built‐in self‐attention mechanism enables it to non‐linearly record relationships between segments of the input sequence, making it a good choice for processing high‐dimensional CT images.^150^ The Transformer model has also shown promise in other fields, such as image processing, and has reached state‐of‐the‐art performance in a variety of natural language processing applications. Additionally, the Transformer model can process each part of the image separately, making it more efficient for handling large images compared to typical convolutional neural networks.

However, there are also some drawbacks to using the Transformer model in CT image denoising. Obtaining good results with the model requires a significant amount of labeled data, similar to other DL models. Additionally, the Transformer model is sometimes viewed as a black box model because of the difficulty in understanding how it generates its predictions. The model's built‐in self‐attention mechanism can also make it hard to determine which features of an image should be prioritized when creating a forecast. Due to these limitations, Transformer‐based models have not gained substantial traction in CT image denoising.

### 4.4 Other methods

VAEs are **DL** models that consist of an encoder network and a decoder network that are trained together to learn a compressed representation of the input data, which can then be used to generate denoised output images.^160^ ResNets utilize residual connections to enable training of much deeper networks. ResNet‐based CT denoising methods use a network architecture that can learn to remove noise while preserving important image features. Attention‐based Networks employ attention mechanisms to selectively focus on important image features while denoising the input image.^72^

## 5 TRAINING, VALIDATION, AND EVALUATION

### 5.1 Training methods

Across the studies reviewed, six distinct learning methods have been employed, including supervised, semi‐supervised, unsupervised, weakly‐supervised, self‐supervised, and self‐attention.

Supervised and unsupervised DL‐based methods are two common approaches for CT image denoising using DL models. Supervised DL‐based methods involve training a model using a set of labeled data, where each input image is paired with a corresponding clean image. The model learns to map noisy images to clean images using this labeled data. Supervised DL‐based methods have been shown to produce high‐quality denoised CT images, and they are effective at removing various types of noise patterns. Out of 99 studies reviewed, 64 studies^42^, ^59^, ^60^, ^61^, ^62^, ^63^, ^64^, ^65^, ^66^, ^67^, ^68^, ^71^, ^72^, ^74^, ^76^, ^77^, ^79^, ^80^, ^83^, ^84^, ^86^, ^88^, ^89^, ^90^, ^91^, ^92^, ^93^, ^97^, ^98^, ^99^, ^100^, ^101^, ^102^, ^103^, ^104^,^106^, ^107^, ^108^, ^109^, ^111^, ^112^, ^113^, ^114^, ^115^, ^116^, ^117^, ^118^, ^119^, ^123^, ^124^, ^125^, ^126^, ^132^, ^134^, ^137^, ^138^, ^139^, ^142^, ^147^, ^153^, ^159^, ^161^, ^162^, ^163^ apply supervised training method. CNNs are a common type of supervised DL‐based model used for CT image denoising.

In contrast, unsupervised DL‐based methods do not require labeled data. Instead, they use techniques such as GANs, VAEs, or self‐supervised learning to estimate the underlying distribution of clean images from the noisy images. Twenty studies^85^, ^87^, ^120^, ^121^, ^122^, ^127^, ^128^, ^129^, ^130^, ^131^, ^133^, ^135^, ^136^, ^141^, ^143^, ^145^, ^146^, ^148^, ^149^,^154^, ^158^ apply different unsupervised training approaches. Unsupervised DL‐based methods rely on the assumption that the noisy image can be modeled as a combination of a clean image and additive noise, and aim to estimate the clean image from the noisy input. These methods are effective in reducing noise levels in CT images, but they may not produce as high‐quality denoised images as supervised methods.

Self‐supervised learning is an unsupervised method that doesn't rely on external labels.^70^, ^82^, ^94^, ^95^, ^105^, ^140^, ^152^ In self‐supervised learning, the model creates its own pseudo‐labels or supervisory signals by designing pretext tasks that require understanding and processing of the data. These pretext tasks are constructed in a way that the model learns meaningful features or representations that can then be transferred to downstream tasks. Once the model learns to solve these pretext tasks, the learned representations can be transferred to the actual target task, often through fine‐tuning or other transfer learning techniques.

Weakly‐supervised learning lies between supervised learning and unsupervised learning. In weak supervision, the labels might be noisy, partial, or coarse, which makes the learning task more challenging. Despite the limited or noisy labeling, the goal is to leverage this weaker supervision to learn meaningful patterns and representations from the data.^75^, ^96^

Different from weakly‐supervised learning, although they both involve working with less‐than‐full supervision. The primary goal of semi‐supervised learning is to leverage the available unlabeled data to improve the model's performance on tasks that require supervised learning. The model uses the labeled data to learn a mapping from inputs to outputs, and it also learns from the unlabeled data to discover patterns and structures that aid in generalization.^144^

Self‐attention is employed in Transformer‐based models.^150^ Self‐attention learning involves calculating attention scores between each pair of elements in the sequence and using these scores to compute weighted sums, which are then used to update the representations of the elements. The key idea is to allow each element to attend to other elements, assigning different degrees of importance to them based on their contextual relevance.

In general, supervised DL‐based methods generally outperform unsupervised DL‐based methods in terms of the quality of the denoised images. This is because supervised methods can learn from labeled data, which provides more information about the underlying noise patterns in the images. However, supervised DL‐based methods require a large amount of labeled data to train, which can be time‐consuming and expensive to obtain.

Unsupervised DL‐based methods are more suitable when labeled data are scarce or unavailable. They are still effective in reducing noise levels in CT images, and they can be faster to implement since they do not require training on labeled data.

### 5.2 Validation methods

Validation methods are used to assess the performance and generalizability of a trained model on new, unseen data. The choice of validation method depends on factors such as dataset size, data distribution, and the model's complexity.

K‐fold cross‐validation is the most popular validation method typically used for supervised learning tasks where there are labeled data and a target variable to predict, including single fold,^68^, ^71^, ^72^ 5‐fold,^97^, ^98^, ^99^, ^100^, ^102^ and 10‐fold cross‐validation.^128^, ^129^, ^130^, ^131^ In general, the performance results from higher iterations can be averaged to obtain a more reliable estimation of the model's performance.

Validation in unsupervised learning is a bit different from supervised learning since there are no ground truth labels to compare predictions against. Instead, the focus is often on assessing the quality of the learned representations or clusters. Techniques such as the gradient‐based Adam optimizer^136^ and 3D dictionary learning iterative reconstruction^148^ have been employed to assess potential overfitting during training. Shan et al.^149^ adopted a cluster stability analysis approach, gauging the robustness of clusters across multiple runs with slight data variations or algorithmic parameter adjustments. Zhang et al.^87^ validated their training method by introducing diverse datasets and modifying experimental setups. Several studies^120^, ^121^, ^122^, ^148^ took a comparative approach by evaluating their methods across different image patches derived from alternate datasets. This multifaceted validation framework underscores the complexity and nuance associated with validating unsupervised learning models in the absence of explicit ground truth.

Remember that validation in unsupervised learning is often more subjective and context‐dependent compared to supervised learning. The choice of validation methods depends on the specific goals of the analysis and the characteristics of the data. It is common to use a combination of methods to gain a comprehensive understanding of the quality of the clustering or learned representations.

### 5.3 Training and validation data

**FIGURE 4:**
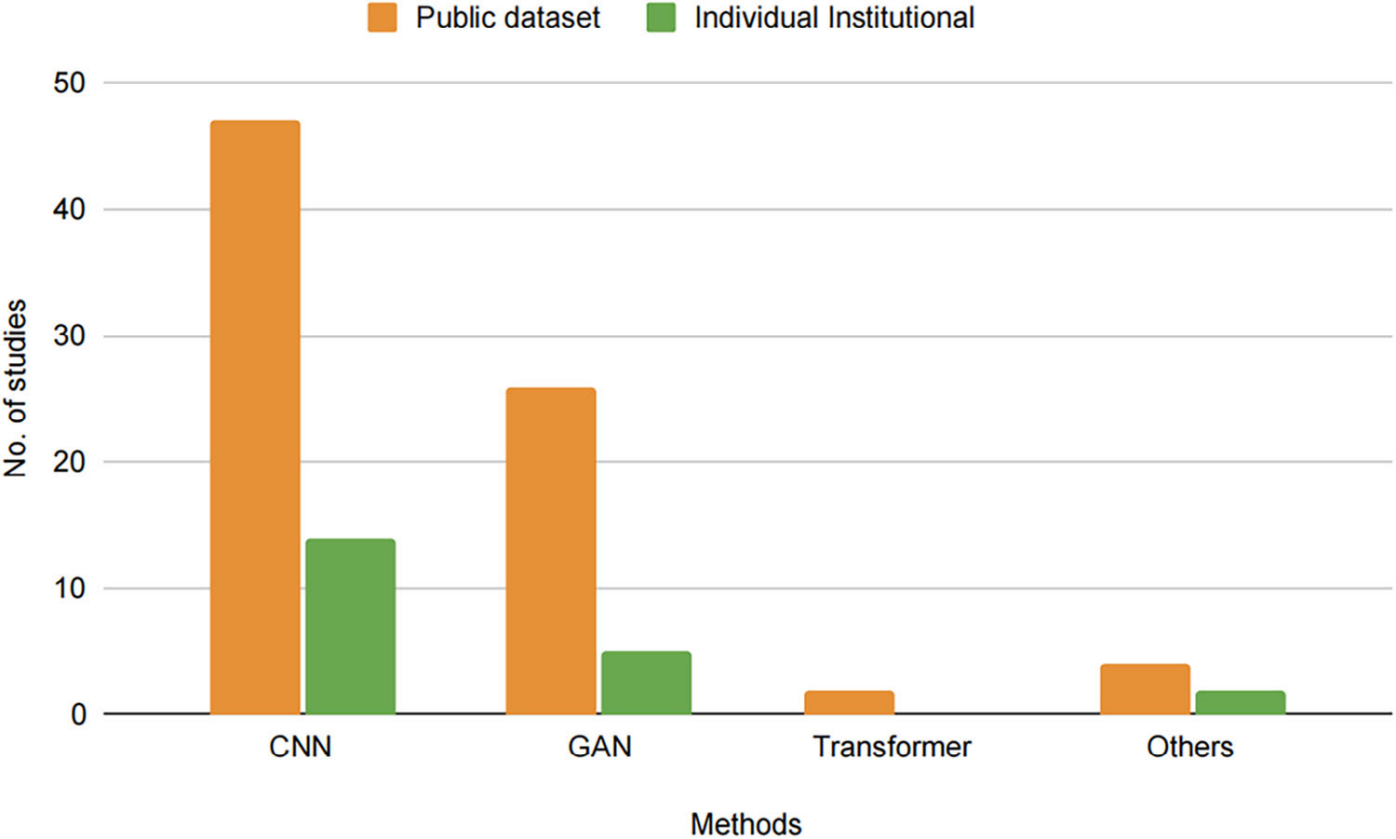
The distribution of datasets employed across the reviewed studies.

DL‐based models for CT image denoising demand substantial quantities of high‐quality training data to achieve satisfactory performance. These datasets are often drawn from both publicly available sources and individual institutional collections. **Figure** **4** illustrates the distribution of datasets employed across the 99 studies surveyed.

Public datasets, such as the NIH Chest CT dataset, can be a valuable resource for training CT image denoising models. These datasets typically contain large numbers of high‐quality images that have been carefully labeled by experts. Using these datasets can save significant time and effort in data collection and annotation, and can also help to ensure that the models are trained on a diverse range of images with a wide range of noise characteristics. **Table** **2** shows a summary of publicly available datasets based on the studies reviewed.

**TABLE 2:** Summary of publicly available datasets the reviewed studies used (links verified as of 12/15/2023).

Public dataset	Data type	Region	No. of studies	Description
NIH AAPM Mayo^122^ link	Low dose CT, routine dose CT	Abdomen, head, chest	36^61^, ^64^, ^65^, ^66^, ^68^, ^71^, ^72^, ^74^, ^75^, ^76^, ^77^, ^82^, ^95^,^106^, ^107^, ^113^, ^114^, ^115^, ^116^, ^119^, ^120^, ^122^,^123^, ^125^, ^129^, ^131^, ^138^, ^139^, ^140^, ^143^, ^144^,^148^, ^149^, ^150^, ^152^, ^153^	5936 NDCT images with 512 × 512 and quarter‐dose simulated LDCT images from 10 patients.
Piglet^127^ link	Low dose CT, routine dose CT	Piglet	5^72^, ^108^, ^127^, ^130^, ^147^	2260 CT images from the piglet dataset as the training set
(ECG)‐gated cardiac CT^136^ link	CT	Cardiac	2^136^, ^157^	13,482 pairs training data of low‐dose CT images
LDCT projection data^151^ link	Low dose CT,	Abdomen, head, chest	3^92^, ^105^, ^151^	Low‐dose projections are achieved by adding Poisson noise to the standard dose projections.
Deep Lesion dataset^121^ link	CT	Renal lesions, bone lesions, pulmonary nodules, and lymphadenopathy	2^62^, ^121^	12 000 images from 3225 patients are used.
TCIA^135^ Link	Low dose CT, routine dose CT	Head	8^63^, ^69^, ^72^, ^90^, ^108^, ^114^, ^117^, ^135^	50 head scans, a total of 1782 routine‐dose (NDCT) and low‐dose (LDCT) slices.
LIDC/IDRI database^126^ link	Low dose CT	Lung, dental	1^126^	280 ground truth train XXX.hdf5 files and 28 ground truth XXX.hdf5 files. Each HDF5 file can be extracted into 128 images
TCGA‐COAD^93^ link	Low dose CT, routine dose CT	Bones	1^93^	200 different CT images with a size of 512 × 512 pixels as training data.
XCAT^96^ link	Low dose CT	Cardiac	1 ^96^	3000 image slices from 10 pediatric XCAT phantoms
ISLES^91^ link	CT	Stroke lesion	1 ^91^	94 volumes of dynamic CT images each containing varying number of axial slices (two to eight) and time‐frames (40–50)

Dataset (public or institutional) that is used for training/validation, independent dataset indicates those different from public and institutional (independent test).

### 5.4 Model evaluation

Evaluating a DL denoising model involves assessing its ability to effectively reduce noise while preserving important image details. Generated denoised images are usually compared against the ground truth images such as FBP,^164^, ^165^, ^166^ IR,^167^, ^168^, ^169^, ^170^ or other DL methods.^158^, ^159^, ^163^, ^171^, ^172^, ^173^, ^174^, ^175^, ^176^, ^177^, ^178^, ^179^, ^180^, ^181^, ^182^ This comparison can provide insights into the model's relative strengths and weaknesses in terms of denoising performance. If the model's performance is not satisfactory, consider iterative improvements such as architecture modifications, hyperparameter tuning, or dataset augmentation.^158^ Repeat the evaluation process to assess the impact of these changes on the denoising performance.

### 5.5 Generalizability

Generalizability is an important consideration when evaluating the effectiveness of DL‐based CT image denoising models. Among 99 papers reviewed, only five studies conducted the independent test.^106^, ^139^, ^156^, ^168^, ^180^ An independent test if a mode can be able to effectively denoise CT images in a variety of contexts is necessary, however, this is not yet realized.

To ensure generalizability, it is important to use a diverse set of training data that includes images from different scanners, protocols, and patient populations. This can help to ensure that the model is able to handle a wide range of variations in image quality and characteristics.

### 5.6 Evaluation metrics

**FIGURE 5:**
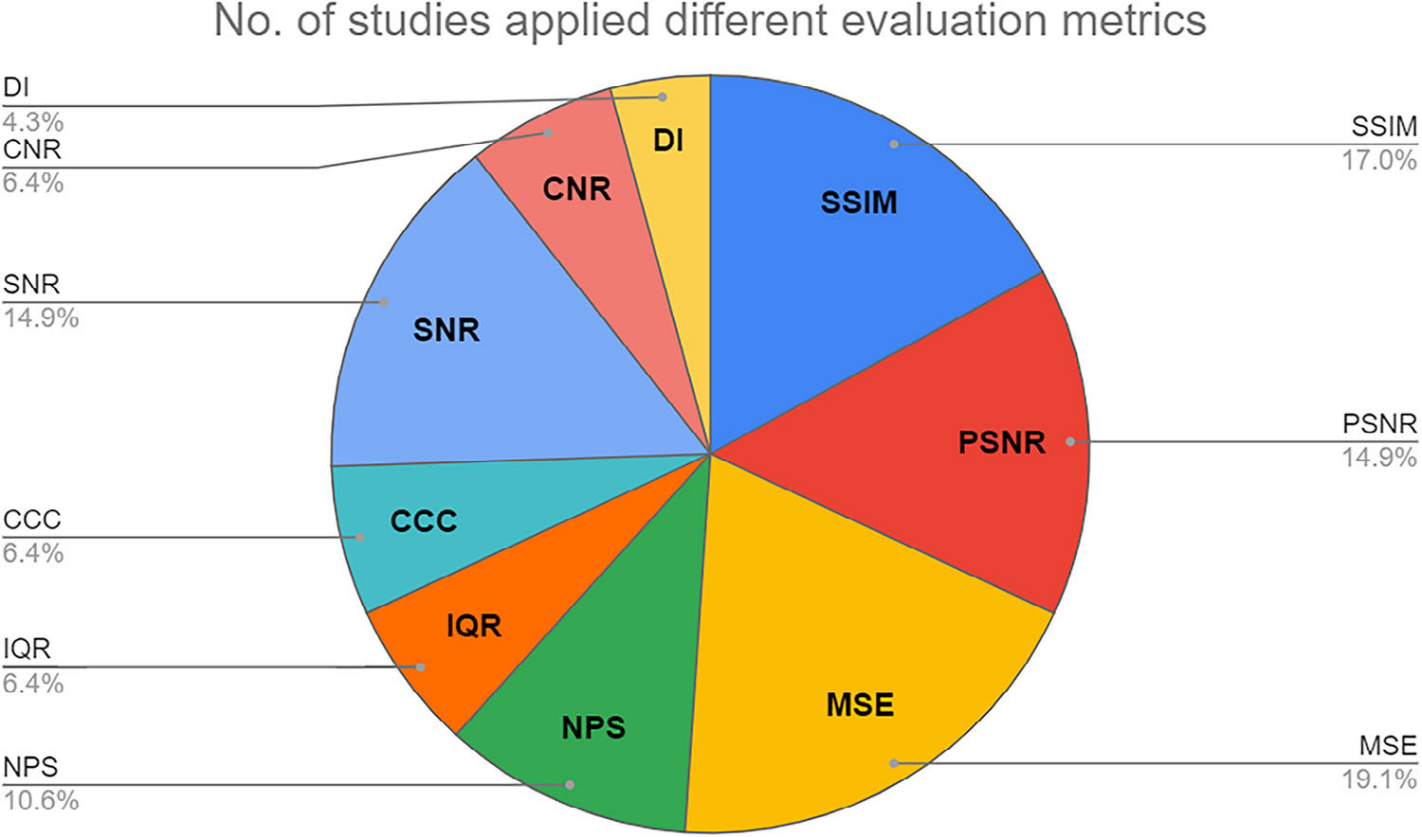
Evaluation metrics and their distribution. CCC, concordance correlation coefficient; CNR, contrast to noise ratio; DI, Dunn's index; IQR, interquartile; MSE, mean square error; NPS, noise power spectrum; PSNR, peak signal noise to ratio; SNR, signal to noise ratio; SSIM, structural similarity index.

Figure 5 illustrates the distribution of various evaluation metrics employed across the surveyed papers. These metrics, including Dunn's Index (DI), Interquartile Range (IQR), Signal to Noise Ratio (SNR), Peak Signal to Noise Ratio (PSNR), Contrast to Noise Ratio (CNR), Mean Square Error (MSE), Noise Power Spectrum (NPS), Concordance Correlation Coefficient (CCC), and Structural Similarity Index (SSIM), offer detailed insights into the quality of image denoising.

DI evaluates the clustering quality by measuring how well similar pixels are grouped, assessing the distinctness of pixel groupings. IQR gauges the spread of pixel values, useful for identifying noise outliers. SNR measures the ratio of signal strength to noise, directly reflecting the impact of noise. PSNR benchmarks the quality of a denoised image against a reference, with higher values indicating superior denoising. CNR focuses on the preservation of contrast amid noise reduction. MSE computes the average of the squared differences between original and denoised images, providing an error estimate. NPS examines the distribution of noise across frequencies, and CCC determines the agreement between the denoised and reference images. SSIM assesses the preservation of structural information, factoring in luminance and contrast.

The common advantages of these metrics lie in their capability to quantify aspects of image quality, from noise level to structural integrity, which is crucial for evaluating denoising algorithms. They serve to analyze both spatial and frequency domains and are versatile in addressing various image fidelity aspects, such as outlier detection and diagnostic preservation.

While a previous study has shown that there is a strong correlation between SSIM and radiologists’ evaluations for diagnostic quality and low‐contrast detectability and a moderate correlation for texture,^167^ these metrics share the limitation of not perfectly mirroring human visual perception. They often involve subjective elements, like image partitioning or region selection, which may not fully encompass the complexity or diagnostic importance of the image content. Some metrics, such as SNR, are straightforward and intuitive, while others, like NPS, may be complex for those without expertise. Moreover, they can be overly sensitive to outliers, might not reflect perceptual nuances, or may not coincide with subjective quality assessments, despite their objective accuracy.

The selection of appropriate evaluation metrics depends on the specific needs and nuances of the image denoising task at hand. Researchers often employ multiple metrics to thoroughly assess the performance of DL denoising algorithms, considering perceptual quality, fidelity to the original image, and detail preservation. Beyond quantitative metrics, subjective assessments are also vital,^183^, ^184^, ^185^, ^186^ as they involve visual inspection for noise reduction, detail preservation, and overall image fidelity, offering insights into potential limitations or artifacts not captured by quantitative measures.

## 6 APPLICATION

The potential application of DL‐based CT denoising models has been identified to enhance image quality, improve diagnostic accuracy and streamline radiological workflows. The majority of research focusing on the objective image quality evaluations of DL algorithms has consistently demonstrated remarkable noise reduction compared to FBP and IR at equivalent or lower radiation dose levels.^74^, ^77^, ^79^, ^82^, ^90^, ^92^, ^93^, ^95^, ^103^, ^104^, ^113^, ^114^, ^147^ Additionally, the implementation of DL for metal artifact reduction demonstrates superior results when compared to IR.^62^, ^86^, ^119^, ^121^

CT image denoising approaches show promising potential, but are not widely accepted in routine clinical practice. To date, three CT vendors have introduced DL‐based reconstruction algorithms: TrueFidelity by GE Healthcare, AiCE by Canon Medical Systems, and Precise Image by Philips Healthcare.^187^ Among these, TrueFidelity and PreciseImage are direct algorithms that reconstruct the sinogram directly into an image, without FBP or IR. While AiCE is an image‐based algorithm that requires either FBP or IR. All three algorithms are based on CNNs and are trained using low‐dose sinograms or images. The practical application of these algorithms has been comprehensively outlined by Koetzier et al,^187^ indicating that they effectively reduce image noise at low radiation doses. Nevertheless, it is important to note that their full integration into clinical practice remains an ongoing process.

## 7 CHALLENGES

### 7.1 Data for model training

One of the primary challenges in developing a DL model for CT image denoising is the availability of high‐quality training data. Publicly available datasets can be a potential source, but it is crucial to carefully evaluate the quality and consistency of the data before using it for model training. Variations in image quality, noise characteristics, and other factors can affect the model's performance, so it is important to ensure that the dataset is representative of the specific clinical applications for which the model will be used.

The amount of data required for training or fine‐tuning a DL model for CT image denoising will depend on the model's complexity and the specific task. In general, larger amounts of high‐quality data will improve the model's generalizability and ability to capture complex patterns in the data. However, obtaining large amounts of medical imaging data can be challenging due to privacy concerns and ethical considerations. In such cases, transfer learning can be a useful approach, where a pre‐trained model on a related task or dataset is fine‐tuned on the target CT image denoising task using a smaller amount of data.

The quality and diversity of the training data are critical factors in achieving optimal results with DL models. Therefore, careful selection and curation of the training data are essential. Additionally, data augmentation techniques, such as rotation, scaling, and flipping, can be used to artificially increase the size of the training dataset and improve the model's robustness. It is essential to comply with relevant ethical and legal guidelines for data sharing and use when using medical imaging data for model training.

### 7.2 Model generalizability

It is important to evaluate the model's performance on a separate validation set that was not used during training. This can provide an indication of the model's ability to generalize to new, unseen data. Other factors that can impact generalizability include the complexity of the model, the amount of training data used, and the specific denoising algorithm employed. It is important to carefully consider these factors when developing and evaluating DL‐based CT image denoising models in order to ensure that they are effective and reliable across a wide range of contexts.

### 7.3 Evaluation metrics

Evaluation metrics play a crucial role in assessing the performance of DL‐based CT denoising models. We review some commonly used evaluation metrics for CT denoising models.

It is important to note that each metric has its own advantages and disadvantages, and no single metric can fully capture the performance of a CT denoising model. It also needs to be noted that the evaluation based on those metrics is not necessarily aligned with radiologists’ evaluation. It is recommended to use a combination of metrics for a comprehensive evaluation of the model's performance.

### 7.4 Vulnerability to adversarial attacks

One challenge for DL models, including those used in CT image denoising, is adversarial attacks. These attacks involve adding carefully crafted noise to the input image to evade the denoising model's filters and produce a degraded output image. In medical imaging applications, misdiagnosis or incorrect treatment resulting from such attacks could have serious consequences.

To address the vulnerability of DL models to adversarial attacks, researchers have proposed several defense mechanisms, including adversarial training, input perturbation, and model compression. These approaches improve the model's robustness to adversarial attacks by incorporating adversarial examples into the training process or modifying the input data to prevent the model from being deceived by adversarial perturbations. While these defenses can reduce the vulnerability of DL models, they are not foolproof and can sometimes introduce new vulnerabilities. Thus, continued research is necessary to develop more robust and reliable defense mechanisms to ensure the safety and effectiveness of DL‐based CT image denoising models.

## 8 PERSPECTIVES

The use of DL models has revolutionized the field of CT image denoising, yielding significant improvements in performance compared to traditional methods. However, there remain several challenges to overcome, such as the need for large amounts of high‐quality training data, potential vulnerability to adversarial attacks, and difficulty in interpreting results.

To further improve the effectiveness and reliability of DL‐based CT image denoising, it is important to continue researching and developing new approaches. One promising avenue is transfer learning, which could improve the generalizability of DL‐based CT image denoising models. Additionally, incorporating explainable AI (XAI) techniques into DL‐based CT image denoising models could help users understand how and why the model is making decisions.

Uncertainty estimation is another important aspect of DL that could be incorporated into CT image denoising models to help users assess the reliability of the model's denoising results. Moreover, real‐time denoising could be especially useful in clinical settings where fast processing is crucial. Future research can focus on optimizing DL‐based CT image denoising models to achieve real‐time performance.

Overall, the future of DL‐based CT image denoising looks promising, with continued advancements and improvements likely to lead to even more effective and reliable denoising methods.

## AUTHOR CONTRIBUTIONS

All listed authors contributed to the literature search and to drafting the manuscript.

## CONFLICT OF INTEREST STATEMENT

The authors have no conflict of interest to declare.
